# An Ogilvie’s syndrome: a rare case of large bowel pseudo-obstruction

**DOI:** 10.1186/s12245-025-00857-9

**Published:** 2025-03-06

**Authors:** Mazen Mohammad, Khaled Alsheikh, Sabet El Madlaji, Muhamad Zakaria Brimo Alsaman

**Affiliations:** 1 General Surgery Department, Abd Al Wahab Agha Hospital, Aleppo, Syria; 2 Orthopedic Surgery Department, Abd Al Wahab Agha Hospital, Aleppo, Syria; 3 Cardiology Department, Aleppo University Hospital, Aleppo, Syria; 4 Faculty of Medicine, University of Aleppo, Aleppo, Syria

## Abstract

**Introduction:**

Ogilvie’s Syndrome (OS) is a rare but serious functional disorder characterized by dilatation of the colon, typically affecting the cecum and right colon, in the absence of any mechanical obstruction.

**Case presentation:**

We present an unusual case of Ogilvie’s Syndrome in a 67-year-old female patient following elective dynamic hip screw surgery. She presented with gradual abdominal distention, as well as gas and stool retention. On the ninth postoperative day, abdominal examination revealed significant distention, marked tympanitic sounds upon percussion, diffuse tenderness on palpation, diminished bowel sounds, and moderate abdominal pain. Investigation tools demonstrated gross dilated in colons, up to 92.4 mm at the cecum level by Abdominal CT which, confirming the diagnosis of Ogilvie’s Syndrome. The team opted for conservative treatment, including nasogastric tube (NGT) insertion, fasting, and intravenous fluids. Subsequent imaging a few days later indicated a reduction in colonic diameter (cecum measuring 38 mm) and an improvement in the patient’s overall condition.

**Conclusion:**

Although Ogilvie’s Syndrome is infrequently encountered, clinicians should maintain a high index of suspicion for gas and stool retention following surgical procedures. It is essential to be familiar with diagnostic methods and management protocols for this condition.

## Introduction

Ogilvie’s Syndrome (OS) is a rare but serious functional disorder characterized by dilatation of the colon, which usually involves the cecum and right colon without any mechanical obstruction. It was first reported in 1948 by Sir William Ogilvie [1].

It occurs in less than 1% of patients undergoing surgery (organ transplant, orthopedic, gynecologic, and urologic surgeries), with increases both morbidity and mortality rates [2, 3].

Abdominal X-ray (AXR) or a CT scan of the abdomen mainly is used in diagnosis. The primary finding is dilatation of the proximal colon, which may occasionally extend to the rectum, without any evidence of mechanical obstruction.

We report a rare case of Ogilvie’s Syndrome in a 67-year-old female following elective dynamic hip screw surgery. She presented with gradual abdominal distention, as well as gas and stool retention. In this manuscript, we aim to highlight the importance of considering this condition after surgery, even though it is rarely seen in clinical practice.

## Case presentation

A 67-year-old, non-alcoholic, non-smoker woman with type 2 diabetes, hypertension, and hyperlipidemia was admitted to our hospital for elective dynamic hip screw surgery due to an intertrochanteric hip fracture. Patient’s BMI: 32 kg/m². We put the patient on opioids for three days after surgery for postoperative pain management.

Two days after the surgery she referred to the general surgery department complaining of gradual abdominal distention, gas and stool retention.

Abdominal examination revealed soft abdominal on palpation, tympanitic on percussion and normal bowel sound.

Vital signs were as follows: Blood pressure: 140/70, Heart rate: 95, SaO_2_: 97, temperature: 38^C^.

Laboratory studies (Table 1):

**Table 1:** Summary of clinical investigations

Investigation	Value	Normal
WBC	11.1 × 10^9^/L	4.5–11.0 × 10^9^/L
HBG	9.7	12.1 to 15.1 g/dL
urea	86	17–43 mg/dL
creatinine	1.5	0.50–1.30 mg/dL
K	3.77	3.5–5.2 mmol/L
Glucose	263	65–110 mg/dl
CRP	0.5	0.3–1.0 mg/dL

Digital exam revealed small amount of stool, trial of enemies relieved a little amount of gas and mucus. We decided to keep the patient in the ward for observation.

**Fig. 1:**
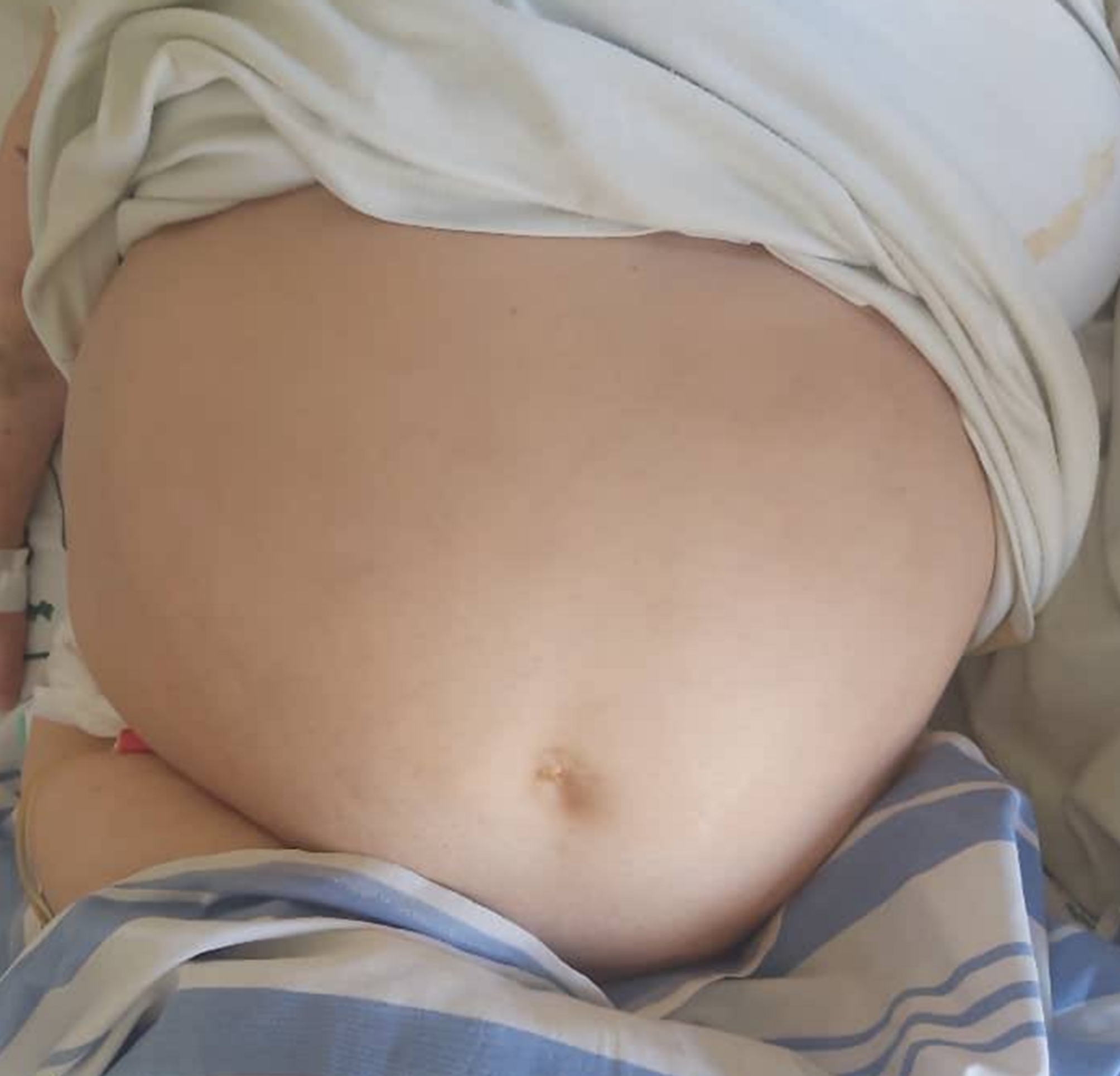
Gross abdominal distention on abdominal examination

Abdominal examination on the ninth day relieved gross abdominal distention with severe tympanitic on percussion, diffuse tenderness on palpation and diminished bowel sound, with moderate abdominal pain (Fig. 1).

We could not perform an abdominal X-ray on the patient due to their immobile status.

**Fig. 2:**
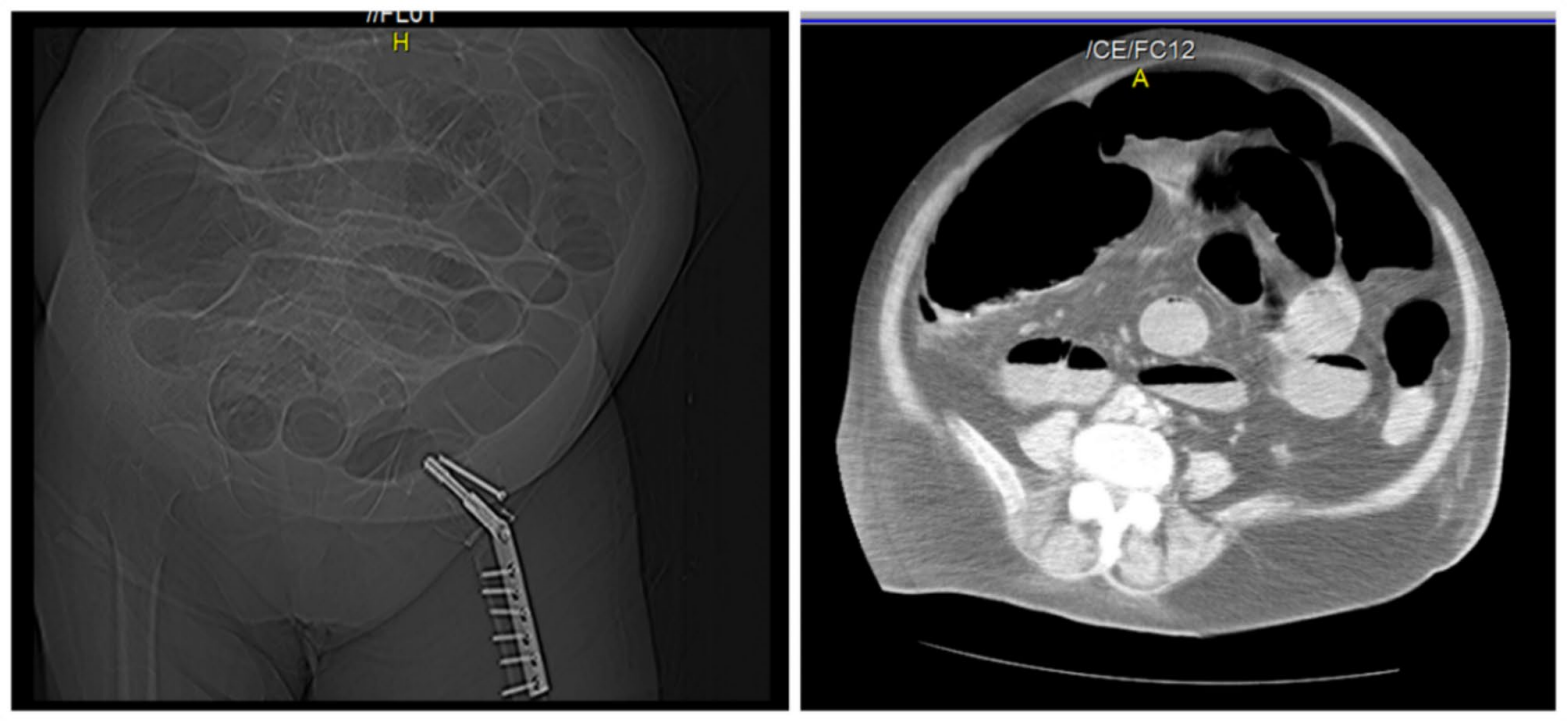
Abdominal CT that showed a gross dilated in colons, up to 92.4 mm at the cecum level

Then we decided to carry an Abdominal CT that showed a gross dilated in colons, up to 92.4 mm at the cecum level (Fig. 2).

A diagnosis of Ogilvie syndrome was made based on CT results and after excluding mechanical and functional causes.

The team decided to continue conservative management with NGT, fasting, replacement for low potassium and IV fluids, since the cecal diameter is less than 12 cm and no signs of complications.

a small amount of fluid was evacuated on NGT.

On the tenth day after surgery, she suffered from hypoxia SaO_2_:85% and was transferred to the intensive care unit for monitoring and improvement of general condition.

**Fig. 3:**
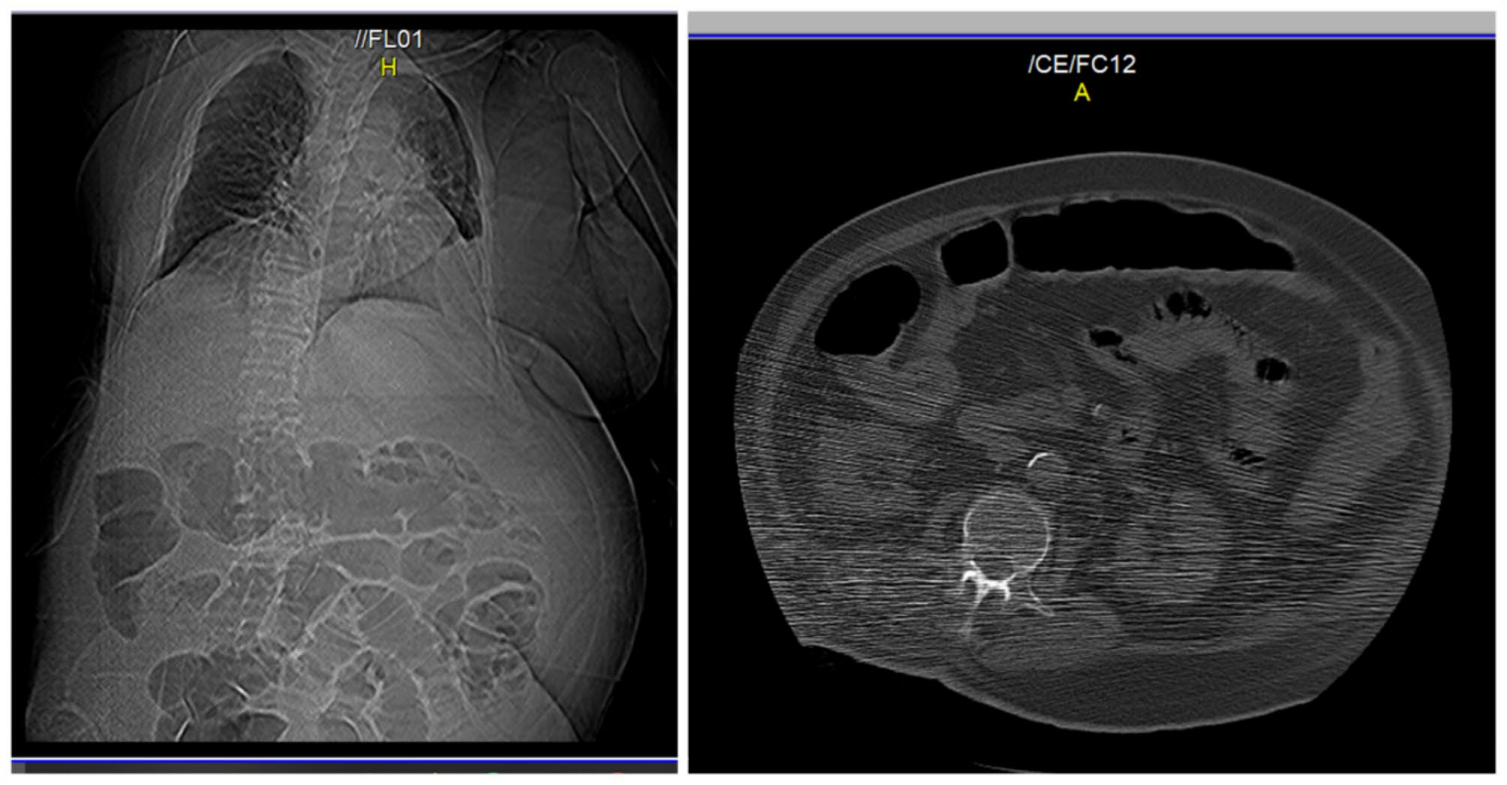
New abdominal CT after the management plan showed decrease in colon diameter

The next day, an abdominal examination showed soft abdomen and slight tympanitic percussion with gas and stool passing. A new abdominal CT scan showed a decrease in colon diameter (cecum 38 mm) (Fig. 3). Two days later the patient was discharged to home with good general condition.

During the three month’s follow-up and review, the patient reported improving in her symptoms.

## Discussion and conclusions

Acute colonic pseudo-obstruction (ACPO) also known as Ogilvie’s Syndrome (OS) is a rare but serious functional disorder characterized by dilatation of the colon, which usually involves the cecum and right colon without any mechanical obstruction. OS is often preceded by a surgical intervention [4].

Sir William Ogilvie first described Ogilvie’s Syndrome in 1948. Although the exact mechanism of the pseudo-obstruction remains unknown, the initial manuscript attributed its occurrence to the interruption of autonomic supply to the colon [1]. This is particularly relevant given the higher probability of OS in cases involving trauma, spinal anesthesia, and pharmacologic agents (such as Opioids) that affect the autonomic nervous system.

In an analysis of 400 cases, Ogilvie’s Syndrome was most commonly reported in patients in the sixth decade with a male predominance. The most common surgeries associated with it are cesarean sections and hip surgeries [5].

Another study conducted by Norwood et al., which reviewed all patients who underwent abdominal imaging after orthopedic surgery over a five-year period, indicated that Ogilvie’s Syndrome (OS) is relatively rare following major orthopedic procedures, occurring in nearly 1% of cases, with the majority involving hip surgeries. Notably, only one case out of 21 hip operations was insertion of a Dynamic Hip Screw (DHS). However, it significantly increases both morbidity and mortality rates [6].

The primary clinical presentation is abdominal distention, typically occurring three to seven days post-surgery, though it can develop within the first 48 h. Other symptoms include abdominal pain, nausea, vomiting, and constipation. Paradoxically, some cases may present with diarrhea. In rare instances, dyspnea has been reported due to colonic distention, which, in our case, necessitated ICU admission.

Diagnosis is confirmed by excluding other causes of intestinal obstruction, which requires abdominal imaging such as an Abdominal X-ray or a CT scan of the abdomen. A contrast enema using a water-soluble contrast is not preferred due to the high risk of perforation and subsequent peritonitis. The primary finding is dilatation of the proximal colon, which may occasionally extend to the rectum, without any evidence of mechanical obstruction [7].

Laboratory findings are typically normal or may show mild leukocytosis and metabolic abnormalities, such as electrolyte imbalances, particularly hypokalemia [8]. Differential diagnoses include mechanical obstruction, and toxic mega colon [9].

Choosing the optimal therapy depends on two main factors: the cecal diameter and the presence of complications. For patients without complications, conservative management is preferred. This primarily involves discontinuing pharmacologic agents especially opioids that reduce intestinal motility and decompressing the intestines using nasogastric tube and enemas to reduce the risk of perforation and peritonitis. Patients should be kept NPO (nothing by mouth) and given IV fluids to correct any electrolyte imbalances that may be present [10].

Alternatively, if the cecal diameter exceeds 12 cm or if conservative treatment fails, the preferred treatment is the administration of neostigmine, an acetylcholinesterase inhibitor. Neostigmine is given intravenously over a period of 5 min to minimize autonomic complications such as bradycardia and bronchoconstriction. Therefore, it is crucial to have atropine or glycopyrrolate on hand [11].

If the patient does not respond to the initial dose of neostigmine, a second dose can be administered after 24 h. A study demonstrated that the clinical response rate ranges from 40 to 100% [12].

Other methods to manage ACPO include colonoscopic decompression, which is reserved for patients who do not respond to neostigmine. This procedure is technically challenging and carries a high risk of complications, such as perforation.

Surgical treatment is reserved for patients with complications such as perforation, ischemia, and peritonitis, as well as those who are refractory to other management regimens [10].

In cases of acute intestinal pseudo-obstruction, the mortality rate is approximately 15% with prompt and appropriate management. However, when complications such as ischemia or perforation occur, the mortality rate can escalate to around 40% [5, 13].

The development and progression of acute pseudo-obstruction may be affected by several factors, including advanced age, electrolyte imbalances, and conditions such as diminished functional status, immobility, diabetes mellitus, non-operative trauma, severe infections, post-myocardial infarction states, and neurological disorders like Parkinson’s disease [14, 15].

This underscores the importance of early diagnosis of ACPO. Early detection allows for timely intervention, significantly improving patient outcomes. In its early stages, ACPO is manageable and can prevent severe complications.

However, if not promptly diagnosed and treated, ACPO can lead to serious complications with high morbidity and mortality rates. This highlights the need for healthcare professionals to maintain a high level of suspicion for this condition, especially in post-surgical patients presenting with symptoms like abdominal distention, pain, nausea, and vomiting.

Although Ogilvie’s Syndrome is rare and not commonly encountered in daily clinical practice, it is important to keep it in mind. Its complications, such as perforation, ischemia, and peritonitis, can be avoided if clinicians are familiar with the diagnostic methods and management algorithms.

## Data Availability

No datasets were generated or analysed during the current study.

## Declarations

### AI and AI-assisted technologies in the writing process

During the preparation of this paper, the authors used the assistance of Microsoft Copilot in order to proofread the manuscript for any grammar and spelling mistakes. After using this tool, the authors reviewed and edited the content as needed and take full responsibility for the content of the publication.
